# Relevance of oxidative stress and inflammation in frailty based on human studies and mouse models

**DOI:** 10.18632/aging.103295

**Published:** 2020-05-27

**Authors:** María Álvarez-Satta, Alejandro Berna-Erro, Estefania Carrasco-Garcia, Ainhoa Alberro, Ander Saenz-Antoñanzas, Itziar Vergara, David Otaegui, Ander Matheu

**Affiliations:** 1 Group of Cellular Oncology, Biodonostia Health Research Institute, San Sebastian, Spain; 2 CIBER of Frailty and Healthy Aging (CIBERfes), Spain; 3 Group of Multiple Sclerosis, Biodonostia Health Research Institute, San Sebastian, Spain; 4 Group of Primary Health, Biodonostia Health Research Institute, San Sebastian, Spain, Health Services Research on Chronic Patients Network (REDISSEC), Spain; 5 Spanish Network of Multiple Sclerosis, Spain; 6 IKERBASQUE, Basque Foundation, Bilbao, Spain

**Keywords:** frailty, aging, biomarker, oxidative stress, inflammation

## Abstract

Frailty represents a state of vulnerability and increases the risk of negative health outcomes, which is becoming an important public health problem. Over recent years, multiple independent studies have attempted to identify biomarkers that can predict, diagnose, and monitor frailty at the biological level. Among them, several promising candidates have been associated with frailty status including antioxidants and free radicals, and also inflammatory response biomarkers. In this review, we will summarize the more recent advances in this field. Moreover, the identification of scales and measurements to detect and quantify frailty in aged mice, as well as the generation of mouse models, have started to unravel the underlying biological and molecular mechanisms of frailty. We will discuss them here with an emphasis on murine models with overexpression of glucose-6-phosphate dehydrogenase and loss of function of superoxide dismutase and interleukin 10, which reveal that altered oxidative stress and inflammation pathways are involved in the physiopathology of frailty. In summary, we provide the current available evidence, from both human cohorts and experimental animal models, that highlights oxidative damage and inflammation as relevant biomarkers and drivers of frailty.

## INTRODUCTION

The population in advanced countries is rapidly aging, and the proportion of people aged 60 and older is forecast to increase from 16% to 26% in 2050 and will triple from 5.8% to 15% in less-developed countries (World Health Organization, 2010). This expansion is due to successful socioeconomic development and public health policies. However, it is also a major socioeconomic challenge as the aging population increases continuously and may jeopardize the sustainability of public health systems. Thus, the group of people older than 65 accounts for over 60% of health care spending in Organization for Economic Co-operation and Development (OECD) countries. In addition, people aged 65 and over present a health care cost per capita five times higher than for those aged less than 65 years.

Aging is accompanied by severe comorbidities, disabilities, and dependency in many cases [1]. Remarkably, age-associated disability is often preceded by frailty. The concept of frailty has evolved considerably in the literature over the last years to develop into a possible consensus. It is a geriatric syndrome defined as the gradual reduction in functional reserve and resilience, as well as impaired adaptive capacity across multiple physiological systems, that increases the vulnerability against stressors and lead to deterioration and adverse health outcomes in the elderly [2]. A further point that is generally agreed is that frailty is a consequence of the process of aging and indeed its prevalence increases with age. Thus, it ranges from 5% to 17% in people of 65 years from different countries; a percentage that progressively increases up to almost 50% in individuals over 85 years-old [3]. Importantly, frailty can precede the development of disability and other adverse events such as falls, fractures, dependence, institutionalization, and death by several years and it is also closely linked to multimorbidity [4, 5]. Indeed, its distribution in aged populations and its role as a risk factor for relevant deleterious outcomes endorses the current consideration of frailty as a priority target for public health [6, 7].

Another factor that remains consistent about frailty is that it is a dynamic syndrome, which can fluctuate between different states of severity and it has potential for reversibility [2]. Indeed, there is increasing evidence that interventions based on cumulative physical activity through moderate exercise or multicomponent intervention (composed by strength, endurance, and balance training) improve function and partially restore a healthy status in frail elders [8–10]. Nutrition intervention studies using more caloric or high-protein diets as well as nutritional supplementation, such as with vitamin D, have also been proposed. These works show promising results with improvements in strength, walking speed, and nutritional status in frail and pre-frail adults, especially when combined with exercise interventions [11], but they still require further validation to become established. Strategies combining interventions based on exercise, nutrition, cognitive training and/or behavioral therapy, as well as those based on comprehensive geriatric assessment, have also been tested resulting in frailty status improvement [12–14]. Despite the positive results with a variety of interventions, there is still the need for additional evidence-based guidelines on how to treat and reverse frailty in patients [14].

Subsequently, there remains an intense debate about how to screen and assess frailty and, indeed, a standard screening instrument for its identification in research and/or clinical practice is currently lacking.

## Frailty measurement

Frailty was first described by Fried and colleagues in the Cardiovascular Health Study (CHS) [15], who established a “frailty phenotype” based on the presence/absence of a set of five criteria: weakness (low grip strength), slowness (slow walking speed), low physical activity, exhaustion (self-reported), and shrinking (unintentional weight loss). According to this definition, an individual is considered as “frail” if three or more of these features are present, whereas he/she is classified as “pre-frail” if having one or two and “robust” when none is recorded. This frailty assessment tool or modifications of it (called “Fried-like” definitions) are therefore mostly based on performance of physical activity criteria.

Another approach to evaluate frailty is the Frailty Index (FI), which considers the accumulation of age-related health deficits displayed by an individual [16]. The FI is calculated as the number of deficits divided by the total number of potential health deficits under consideration; high values of FI indicating a greater degree of frailty. It is important to note the FI not only takes into account physical measures but it also encompasses additional variables including cognitive and psychosocial aspects that allow professionals to evaluate elderly individuals in a more comprehensive manner.

The “Fried´s frailty phenotype” and the FI are the main methods for screening frailty in humans and also in mice as will be presented later. Although they have become seminal to the frailty research field, assessing frailty using these two scales is complicated mainly in primary care settings but also in other clinical settings due to the required techniques and amount of information or time required. Consequently, they are not implemented in health systems, and other scales and indices have been developed in the last 20 years aimed at identifying and screening the condition of frailty in the clinical practice. These can be grouped in accordance with their conceptual approach. One set of tools is based on the measurement of individual´s functional performance, which could resemble some aspects of physical frailty, and includes Gait Speed (GS), Timed Up and Go (TUG) and the Short Physical Performance Battery (SPPB) tests, among others. Another set is composed of tools and questionnaires on the basis of clinical data, routine, and functional performance criteria that are able to predict the occurrence of adverse effects and includes the “FRAIL” scale [17] or the Tilburg Frailty Indicator [18]. Finally, there is a smaller group of measures based on clinical judgment, such as the Clinical Global Impression of Change in Physical Frailty instrument (CGIC-PF) [19], Clinical Frailty Scale [20], or the Gérontopôle Frailty Screening Tool (GFST) [21]. However, as indicated above, there is no agreement on a standard assessment instrument for identification and screening of frailty in research and/or clinical practice. The “FRAIL” scale, which is based on a short five question assessment regarding fatigue, resistance, aerobic capacity, illnesses, and loss of weight, has been validated in several cohorts and will likely become the first consensus tool for frailty so far [2].

Aiming to complement scales, functional tests, and questionnaires, many studies have been conducted to identify frail individuals at biological level. In this regard, several molecules have been proposed, but only a few of them were assessed as clinical biomarkers of frailty, with controversial results [22–24]. This is probably a consequence of the evolution in the conceptualization of frailty, the lack of a standard tool to screen and identify it, and the absence of longitudinal studies, as well as the existence of only a few *omics*-based studies that have compared frail and robust individuals from an unbiased and global perspective.

## Multidimensionality of frailty

One of the characteristics that has reached consensus about frailty is that it represents a multidimensional syndrome, which undoubtedly makes the identification and assessment of frailty more difficult. Thus, there is a clear physical component of frailty; however, increasing attention is being recently paid to additional aspects, mainly cognitive [25, 26] but also additional ones such as psychosocial or nutritional factors [27–30]. Future work will need to establish these domains properly, find tools to assess them specifically, and also demonstrate to what extent these factors play a role in frailty development.

In the next section of this review, we will present a brief overview of the two main domains that have been best described in relation to frailty: the physical and the cognitive ones.

### Physical frailty

The original operational definition of frailty was mainly based on the physical status of individuals [15]. Indeed, Fried and colleagues established a “frailty phenotype” based on the presence of three or more of criteria that included unintentional weight loss, exhaustion, low physical activity, slowness, and weakness as abovementioned. Weight loss has been recently shown to be the last of those five characteristics to manifest [31], which decreases the possibility to improve physical functioning and reverse frailty status.

Physical frailty is described as a medical syndrome with multiple causes and contributors. It is characterized by diminished strength, endurance, and reduced physiological function, and therefore increases the individual’s vulnerability to become dependent and also the risk of death [2]. Physical frailty has been related to disability and sarcopenia, which frequently overlap in clinical practice and can make an accurate diagnosis and management very confusing [32, 33]. However, they are clinically and conceptually distinct, particularly in the case of disability [34]. Indeed, physical frailty can be considered as a pre-disability stage with disability defined as needing assistance with basic activities of daily living [33].

An outstanding characteristic of physical frailty is the loss of muscle quantity and function, which is also part of the definition of sarcopenia and indeed both have physical function impairment as a core component [35]. The lack of standardized definitions means that sometimes both concepts are used interchangeably. However, there is evidence that frailty (according to Fried´s criteria) and sarcopenia (with three specific assessment criteria) are distinct, although related, conditions that often co-exist [36]. This is in line with the idea that sarcopenia may be a component of frailty, but frailty is more multifaceted than sarcopenia alone [2].

### Cognitive frailty

The concept of frailty has been broadened in the last years and it is considered a multidimensional syndrome with multisystem dysfunctions including impaired cognition, in addition to physical impairment, existing interactions among them. This is based on epidemiological evidence that indicates that physical frailty could increase the risk of future cognitive impairment and that cognitive decline may increase the chances to develop frailty [37, 38]. The mutual influences between age-associated cognitive impairment and frailty led to the proposal of the concept of cognitive frailty, which had not been conceptualized until recently. In 2013, an international consensus group from the International Academy of Nutrition and Aging (IANA) and the International Association for Gerontologists and Geriatrics (IAGG) reached a consensus and thereby defined cognitive frailty as a heterogeneous clinical condition described by the simultaneous presence of cognitive impairment and physical frailty [25]. This condition is related to age, and its diagnostic criteria include the presence of mild cognitive impairment (stated by a Clinical Dementia Rating-CDR score of 0.5), physical frailty (based on Fried´s criteria), and the exclusion of Alzheimer´s disease (AD), additional dementias, or pre-existing brain disorders [25].

As occurs with physical frailty, there are no validated tools to accurately detect cognitive frailty in research and clinical practice. The available tests used to evaluate and screen cognitive frailty explore memory performance and executive functions, in addition to other cognitive functions, and include the Mini-Mental State Examination (MMSE), the Montreal Cognitive Assessment test (MoCA), the Alzheimer’s Disease Assessment Scale-cognitive subscale (ADAS-Cog), and speed processing tests [39, 40].

The links between physical and cognitive frailty are strong and there is evidence that both could share risk factors as well as common molecular and pathophysiological mechanisms such as oxidative damage and chronic inflammation [41] or vascular disease [37]. Additionally, both have been associated with hormonal deregulation, gut microbiota, cardiovascular risk, and psychological factors [42–44].

An important characteristic of cognitive frailty is also its potential for reversibility, which could allow the implementation of interventions for improving or restoring cognitive function, and distinguishes this condition from neurodegenerative diseases [39, 44]. However, it cannot be excluded that cognitive frailty, under some circumstances, may facilitate or become a precursor of neurodegenerative processes.

## Biomarkers of frailty in human cohorts: relevance of oxidative and inflammatory contexts

Biomarkers are highly valuable tools in the diagnosis and stratification of patients as well as in the understanding of the molecular and genetic pathways that are dysregulated in disease. Recent years have seen multiple attempts to identify accurate and sensitive frailty biomarkers, while increasing the knowledge about the molecular basis underlying this syndrome. The mechanisms leading to frailty involve several systems, which mainly include oxidative stress and inflammation pathways, but also involve the endocrine, immune, and vascular systems, and consequently, the most promising biomarkers are related to these processes [22, 24]. However, a specific frailty biomarker that can differentiate this syndrome from longevity or other age-associated diseases has not yet been identified and there is not current consensus regarding a panel of biomarkers that could screen frailty in clinical practice [24, 45, 46]. In this review, we will present an update about the sets of molecules related to oxidative stress and inflammation currently considered as potential biomarkers of frail individuals and their putative association with the different components of frailty syndrome.

### Biomarkers of oxidative stress

Reactive oxygen species (ROS) are active molecules generated during enzymatic reactions that play a physiological role under controlled conditions. However, they can generate undesired damaging and oxidizing effects with molecules such as proteins, DNA, and lipids. The intensity of this redox activity is termed “oxidative stress” and an abnormally increased oxidative stress is believed to be a major pathophysiological mechanism underlying disease and aging.

Significant changes in oxidative biomarkers have been detected among robust and frail groups [47]. Most available studies show an increase in oxidative indicators and reduced levels in antioxidant micronutrients in frail individuals (Table 1). Moreover, current knowledge points to a role of abnormal oxidative stress levels in the development of frailty, which enabled the formulation of "The free radical theory of frailty" [48].

**Table 1:** Main biomarkers of oxidative stress associated with human physical and cognitive frailty.

**BIOMARKER**	**TREND (FRAIL *VS*. ROBUST)**	**SAMPLE**	**MEDIAN/MEAN IN FRAIL GROUP**	**MEDIAN/MEAN IN ROBUST GROUP**	**PATIENTS (total)**	**SETTING (country)**	**FRAILTY CRITERIA**	**AUTHOR, YEAR (STUDY)**
Lp-PLA2	Increasing	Blood	median [pM]: 210*	median [pM]: 199	Frail: 142 Pre-frail: 864 Robust: 913 (1,919)	Community (USA)	Frailty phenotype (Fried et al., 2001)	Liu et al., 2016 (Framingham Offspring Study)
8-isoprostane	Increasing	Urine	median [mg/L]: 11.5*	median [mg/L]: 9.5	Frail: 142 Pre-frail: 864 Robust: 913 (1,919)	Community (USA)	Frailty phenotype (Fried et al., 2001)	Liu et al., 2016 (Framingham Offspring Study)
		Urine	mean ± SD [mg/g Cre]: 342 ± 175***	mean ± SD [mg/g Cre]: 235 ± 98	Frail: 34 Pre-frail: 62 Robust: 44 (140)	Outpatients (Japan)	Frailty phenotype (Fried et al., 2001)	Namioka et al., 2017
8-OHdG	Increasing	Serum	median [mg/L]: 2.5*	median [mg/L]: 1.0	Frail: 21 Pre-frail: 56 Robust: 13 (90)	Community and outpatients (Taiwan)	Frailty phenotype (Fried et al., 2001)	Wu et al., 2009
		Urine	mean ± SD [pg/g Cre]: 5.39 ± 2.23***	mean ± SD [pg/g Cre]: 3.90 ± 1.67	Frail: 34 Pre-frail: 62 Robust: 44 (140)	Outpatients (Japan)	Frailty phenotype (Fried et al., 2001)	Namioka et al., 2017
d-ROM	Increasing	Plasma	median [U.CARR]: 371.6***	median [U.CARR]: 339.6	Frail: 210 Pre-frail: 1,463 Robust: 845 (2,518)	Community (Germany)	Frailty phenotype (Fried et al., 2001)	Saum et al., 2015 (ESTHER Cohort Study)
		Plasma	mean ± SD [U.CARR]: 485 ± 86***	mean ± SD [U.CARR]: 418 ± 65	Frail: 34 Pre-frail: 62 Robust: 44 (140)	Outpatients (Japan)	Frailty phenotype (Fried et al., 2001)	Namioka et al., 2017
Protein carbonyls	Increasing	Plasma	mean ± SD [a.u.]: 77.60 ± 15.60*	mean ± SD [a.u.]: 64.36 ± 14.29	Frail: 54 Pre-frail: 278 Robust: 410 (742)	Community (Spain)	Frailty phenotype (Fried et al., 2001)	Inglés et al., 2014 (Toledo Study for Healthy Aging)
MDA	Increasing	Plasma	NA**	NA	Frail: 43 Pre-frail/Robust: 19 (62)	Outpatients (Italy)	Frailty phenotype (Fried et al., 2001)	Serviddio et al., 2009
	Increasing	Plasma	median ± SD [μM]: 3.28 ± 2.45***	median ± SD [μM]: 2.11 ± 1.80	Frail: 54 Pre-frail: 278 Robust: 410 (742)	Community (Spain)	Frailty phenotype (Fried et al., 2001)	Inglés et al., 2014 (Toledo Study for Healthy Aging)
TTL	Reduction	Plasma	median [μM]: 302.9***	median [μM]: 342.1	Frail: 210 Pre-frail: 1,463 Robust: 845 (2,518)	Community (Germany)	Frailty phenotype (Fried et al., 2001)	Saum et al., 2015 (ESTHER Cohort Study)
BAP	Reduction	Plasma	mean ± SD [μmol/L]: 2,390 ± 680*	mean ± SD [μmol/L]: 2,599 ± 627	Frail: 34 Pre-frail: 62 Robust: 44 (140)	Outpatients (Japan)	Frailty phenotype (Fried et al., 2001)	Namioka et al., 2017
VITAMIN E (α-tocopherol)	Reduction	Plasma	median [μM]: 26.7*	median [μM]: 29.6	Frail: 54 Pre-frail: 313 Robust: 460 (827)	Community (Italy)	Frailty phenotype (Fried et al., 2001)	Ble et al., 2006 (InCHIANTI study)
		Plasma	median [μM]: 27.6***	median [μM]: 29.0	Frail: 290 Robust: 1,034 (1,324)	Community and outpatients (France, Spain, Italy)	Frailty phenotype (Fried et al., 2001)	Pilleron et al., 2019 (FRAILOMIC initiative)
		Serum and buccal mucosal cells	median (IQR) [μmol/L]: 26.6 (22.5-32.1)*	median (IQR) [μmol/L]: 27.8 (23.6-32.6)	Frail: 199 Robust: 1,628 (1,944)	Community (14 European countries)	Tilburg Frailty Indicator (Gobbens et al., 2010)	Rietman et al., 2019 (MARK-AGE study)
α-carotene	Reduction	Serum and buccal mucosal cells	median (IQR) [μmol/L]: 0.10 (0.05-0.16)**	median (IQR) [μmol/L]: 0.15 (0.09-0.25)	Frail: 64 Robust: 1,628 (2,128)	Community (14 European countries)	Frailty phenotype (Fried et al., 2001)	Rietman et al., 2019 (MARK-AGE study)
β-carotene	Reduction	Serum and buccal mucosal cells	median (IQR) [μmol/L]: 0.39 (0.26-0.55)*	median (IQR) [μmol/L]: 0.58 (0.37-0.88)	Frail: 64 Robust: 1,628 (2,128)	Community (14 European countries)	Frailty phenotype (Fried et al., 2001)	Rietman et al., 2019 (MARK-AGE study)
β-cryptoxanthin	Reduction	Serum and buccal mucosal cells	median (IQR) [μmol/L]: 0.15 (0.07-0.37)***	median (IQR) [μmol/L]: 0.22 (0.12-0.38)	Frail: 199 Robust: 1,628 (1,944)	Community (14 European countries)	Tilburg Frailty Indicator (Gobbens et al., 2010)	Rietman et al., 2019 (MARK-AGE study)
zeaxanthin	Reduction	Serum and buccal mucosal cells	median (IQR) [μmol/L]: 0.036 (0.022-0.054)***	median (IQR) [μmol/L]: 0.044 (0.028-0.065)	Frail: 199 Robust: 1,628 (1,944)	Community (14 European countries)	Tilburg Frailty Indicator (Gobbens et al., 2010)	Rietman et al., 2019 (MARK-AGE study)

Lp-PLA2: lipoprotein-associated phospholipase A2; 8-OHdG: 8-hydroxy-2'-deoxyguanosine; d-ROM: derivatives of reactive oxygen metabolites; MDA: malondialdehyde; TTL: plasma total thiol; BAP: biological antioxidant potential; NA: not available; SD: standard deviation; U.CARR: Carratelli units; a.u.: arbitrary units; IQR: interquartile range. Asterisks represent *p*-value (frail compared with robust individuals): *p<0.05; **p<0.01; ***p<0.001. Only statistically significant results were included.

### Oxidative stress in physical frailty

One of the molecules proposed as oxidative stress biomarker in frailty is the lipoprotein-associated phospholipase A2 (Lp-PLA2) also known as platelet-activating factor acetylhydrolase. This enzyme belongs to the A2 phospholipase family and catalyzes the hydrolysis of phospholipids to pro-inflammatory or pro-atherogenic lysophospholipids and fatty acids. Interestingly, an association between increased Lp-PLA2 expression in blood and frailty was found in a cohort of 1,919 individuals (aged ≥60 years; 913 fit, 864 pre-frail, and 142 frail) from the Framingham Offspring Study [49]. However, the frail group presented an increased incidence of cardiovascular diseases and this elevation could not be specifically associated with frailty, since Lp-PLA has been also proposed as a predictor of cardiovascular disease and ischemic stroke [50].

Another postulated biomarker of oxidative stress is 8-isoprostane, a prostaglandin-like compound that constitutes a biologically active metabolite produced by the non-enzymatic peroxidation of arachidonic acid. The function of 8-isoprostane ranges from vasoconstriction to modulation of blood platelet aggregation, and the level of 8-isoprostane rises with age [51]. Remarkably, the Framingham Offspring Study found a significant correlation between an increased level of urinary 8-isoprostane and frailty [49]. Similar results were reported within a group of 140 Japanese patients diagnosed with AD (aged ≥65 years; 44 fit, 62 pre-frail, and 34 frail) [52]. However, 8-isoprostane alone cannot be considered as an specific marker of frailty since it has been also proposed as marker of several diseases related to aging such as obesity, lung damage by tobacco, familial hypercholesterolemia, AD, and asthma [53].

Other example is the 8-hydroxy-2'-deoxyguanosine (8-OHdG), a product of oxidative DNA damage routinely used to evaluate DNA integrity and mutagenesis under oxidative stress on several diseases and aging. A significant increase of 8-OHdG was found in frail elders within a group of 90 participants selected from the Chinese population (aged ≥65 years; 13 fit, 56 pre-frail, and 21 frail) [54]. Remarkably, a similar increase was found in the frail group from the Japanese cohort mentioned above [52].

Other compounds suggested as potential biomarkers of oxidative stress in frailty are the derivatives of reactive oxygen metabolites (d-ROM), which measure the total oxidant capacity that is mainly due to hydrogen peroxide levels detected by oxidization of chromogenic substrates [55]. Thus, a total of 2,518 German participants (aged ≥50 years; 845 fit, 1,463 pre-frail, and 210 frail) from the ESTHER Cohort Study were examined and increased d-ROM levels were strongly associated with frailty [56]. A significant increase for d-ROM was also found among the frail group from the Japanese cohort described above [52]. However, d-ROM has also been proposed as an indicator of cardiovascular disease and it has been related to all causes of mortality in elders as well [57], therefore excluding it as a selective biomarker of frailty.

The level of carbonylated circulating proteins, well-established as an indicator of protein oxidative damage, constitutes another candidate for oxidative stress biomarker in frailty. Thus, increased plasma levels of protein carbonylation have been associated with frailty, but not to age or sex, in a sample of 742 participants (aged 65–95 years; 410 fit, 278 pre-frail, and 54 frail) from the Toledo Study for Healthy Aging (TSHA) cohort [58]. Interestingly, previous studies on older women from the Women's Health and Aging Study I (WHAS I) cohort showed that high serum protein carbonyl levels also correlated with poor grip strength [59] and constituted an independent predictor of decline in walking speed and progression to severe walking disability [60]. Moreover, a study performed on institutionalized individuals older than 65 years, with variable degree of dependence based on their Barthel Index, has proposed protein carbonyls as the most accurate marker for severe dependence among other 11 oxidative stress markers [61].

Other markers of oxidative stress have been proposed as biomarkers of frailty, although with contradictory results. For instance, the metabolite malondialdehyde (MDA), which is generated as a consequence of lipid peroxidation and whose presence in serum is indicative of lipid oxidative damage [62]. Plasma MDA levels were found significantly increased in frail elders from the TSHA cohort independent of sex or age [58] and also in a sample of 62 Italian elderly outpatients [63]. However, other studies found no significant differences between frail and non-frail groups [64], and indeed, MDA seems to correlate well with age, indicating that it might be a good marker of aging [65].

With regard to antioxidants, thiols such as glutathione, cysteine, and homocysteine, represent the majority of overall antioxidant mechanisms that protect organisms against oxidative damage. The plasma total thiol (TTL) assay is frequently used to evaluate the physiological antioxidant capacity and consists of determining free thiol groups in serum proteins by their interaction with the chromogen 5,5′-dithiobis(2-nitrobenzoic acid) (DTNB) [66]. The study on German individuals from the ESTHER Cohort Study found a significant decrease of TTL in the frail group, indicating a reduction in the overall antioxidant capacity among frail individuals and suggesting this test as a good indicator of frailty condition [56]. It is also interesting to mention the results obtained from the biological antioxidant potential (BAP) test, designed to assess the global antioxidant activity of blood plasma and based on the idea that oxidative damage occurs when ROS activity exceeds the physiological antioxidant capacity. Thus, two recent studies measured BAP capacity. One of them found significantly lower BAP levels in Japanese frail elders [52], while the study performed in German participants selected from the ESTHER Cohort Study did not found significant differences [56], suggesting that additional studies need to be completed to confirm the efficacy of the BAP test. Another recent paper shows that the mRNA expression of several antioxidants is decreased in frail individuals [67], supporting the idea that a weakened antioxidant defense plays a role in frailty.

On the other hand, vitamins and carotenoids are micronutrients that exhibit a remarkable antioxidant activity, especially in the case of fat-soluble vitamins A and E. The association between decreased levels of these compounds and frailty exists but it is still controversial. For instance, α-tocopherol (the most commonly absorbed form of vitamin E in humans) is converted to α-tocopheroxyl in the presence of ROS, so a decreased amount of vitamin E implies a high conversion activity and therefore high oxidative stress. Two studies found a significant association between low circulating vitamin E levels and frailty: one based on 827 Italian participants (aged ≥65 years; 460 fit, 313 pre-frail, and 54 frail) from the Invecchiare in Chianti study (InCHIANTI) cohort [68] and other performed on 1,324 older adults 65 years of age and older from Approche Multidisciplinaire Intégrée (AMI) (Gironde, France), Three-City (Bordeaux, France), TSHA (Toledo, Spain), and InCHIANTI (Tuscany, Italy) cohorts as part of the FRAILOMIC initiative [69]. Interestingly, both reports found a significant reduction of circulating vitamin E among individuals of the frail group, indicating an increased oxidative stress among frail individuals. However, other analyses focused on elderly women from the WHAS I cohort [64, 70] or participants recruited from Geriatric Day programs [64, 70] did not find any correlation between changes in vitamin E and frailty. Similar inconsistent results were found in studies for vitamin A and other antioxidants such as carotenoids [70, 71], although recent data from the European study to establish biomarkers of human ageing (MARK-AGE) study shows that physical frailty is significantly associated with lower levels of carotenoids such as α-carotene, β-carotene and β-cryptoxanthin [72]. In this sense, it should be noted that serum vitamin levels can be easily altered with diet or during disease, facts that might complicate the interpretation of these results.

### Oxidative stress in cognitive frailty

The identification of molecular markers for cognitive decline in the context of frailty is still very much in its early stage. Among them, recent studies have detected the link between cognitive frailty and oxidative stress. Thus, the MARK-AGE study has recently found that lower levels of two carotenoids (i.e.*,* zeaxanthin and β-criptoxanthin) were associated with *cognitive frailty* and could predict the risk of developing frailty at cognitive level [72]. Furthermore, the same study also suggested that a decreased level of α-tocopherol is a biomarker for cognitive frailty. Levels of MDA and protein carbonyls have been associated with cognitive frailty as well [41]. Additional results obtained from the MARK-AGE study highlight the important role of oxidative stress in the pathophysiology of frailty. Indeed, several carotenoids were independently identified as specific biomarkers for physical and cognitive frailty, as well as for psychosocial frailty.

### Biomarkers of inflammation

The development of a low-grade, chronic pro-inflammatory status in the elderly (i.e., inflammaging) is a physiological condition widely associated with aging and, more recently, also with the pathophysiology of frailty [73, 74].

### Inflammation in physical frailty

The first experimental evidence that linked inflammation to frailty come from a pilot study of Leng and colleagues (2002), who found higher serum levels of interleukin 6 (IL-6) in frail individuals within a group of community-dwelling elders [75]. In the same year, findings from the CHS cohort showed that several pro-inflammatory markers such as C-reactive protein (CRP), factor VIII, and fibrinogen were elevated in frail compared to robust older adults, which supports the role of inflammation in the pathophysiology of frailty [76]. Remarkably, these findings have been validated in several cohort studies to date [77–79] and also for pre-frail individuals that show increased IL-6 and CRP [79]. Interestingly, Darvin and colleagues (2014) using a proteomics-based screening approach reported that higher levels of IL-6 and inflammatory glycoproteins such as transferrin and fibrinogen, but not haptoglobin, were associated with the frail group [78]. Moreover, IL-6 and hemoglobin or hematocrit were inversely correlated in frail patients vs. non-frail individuals, which links inflammation and anemia with frailty [75]. Furthermore, elevated levels of cytokines, especially in the case of IL-6, lead to accelerated protein catabolism and also induce the synthesis of acute-phase proteins such as CRP, haptoglobin, fibrinogen, or factor VIII, which could identify frail and pre-frail individuals [79]. Additional inflammatory markers such as the C-X-C motif chemokine ligand 10 (CXCL10) have been also associated with frail individuals [80, 81]. Apart from classical inflammatory markers, other molecules have been assessed in recent years. Such is the case of neopterin, a catabolic product of guanosine triphosphate (GTP) that is produced by human monocyte-derived macrophages and considered a sensitive indicator of immune activation and pro-inflammatory status. Serum concentration of neopterin is increased with age, so elevated levels have been associated with high risk of frailty and mortality in elderly people from a cohort of 133 individuals [82]. The association between high neopterin levels and frailty was independent of IL-6 levels, suggesting potential monocyte/macrophage-mediated immune activation in frailty status [82].

### Inflammation in cognitive frailty

The role of inflammation in the development of cognitive frailty remains poorly understood and only a few studies have addressed it. Thus, individuals with cognitive frailty presented high levels of inflammatory cytokines such as IL-6, tumor necrosis factor alpha (TNF-α), interleukin 18 (IL-18), interleukin 1 beta (IL-1β), and/or CRP [41, 83], but decreased levels of hematological and endocrinal markers, and poorer nutrition [41]. Moreover, a recent study linked a high inflammatory state (established as serum fibrinogen levels greater than 339 mg/dL) in individuals with “potentially reversible cognitive frailty” (i.e., physical frailty plus mild cognitive impairment) with an increased risk to progress to disability [84]. Notably, inflammation and oxidative stress constitute biological processes that are strongly interconnected. Thus, ROS production by the immune system leads to increased levels of pro-inflammatory cytokines, whereas inflammation triggers cellular side effects, such as generation of free radicals and subsequent oxidative damage, and also the depletion of antioxidants. In summary, these studies confirm that high levels of cytokines and other inflammatory markers in blood are considered as potential biomarkers of different frailty domains (Table 2), suggesting that physical and cognitive frailty might share some biological pathways and highlighting the relevance of inflammation in frailty in the clinical setting.

**Table 2:** Main biomarkers of inflammation in human frailty.

**BIOMARKER**	**TREND (FRAIL *VS*. ROBUST)**	**SAMPLE**	**MEDIAN/MEAN IN FRAIL GROUP**	**MEDIAN/MEAN IN ROBUST GROUP**	**PATIENTS (total)**	**SETTING (country)**	**FRAILTY CRITERIA**	**AUTHOR, YEAR (STUDY)**
IL-6	Increasing	Serum	mean ± SD [pg/mL]: 4.4 ± 2.9*	mean ± SD [pg/mL]: 2.8 ± 1.6	Frail: 11 Robust: 19(30)	Community (USA)	Frailty phenotype (Fried et al., 2001)	Leng et al., 2002
		Serum	mean ± SD [pg/mL]: 0.6 ± 0.98**	mean ± SD [pg/mL]: 0	Frail: 234 Pre-frail: 1,854 Robust: 738 (2,826)	Community (USA)	Frailty phenotype (Fried et al., 2001)	Barzilay et al., 2007 (Cardiovascular Health Study)
		Serum	mean ± SD [pg/mL]: 3.0 ± 1.6*	mean ± SD [pg/mL]: 1.6 ± 1.3	Frail: 16 Robust: 16 (32)	Community (USA)	Frailty phenotype (Fried et al., 2001)	Qu et al., 2009
		Serum	mean ± SD [pg/L]: 2.60 ± 1.63**	mean ± SD [pg/L]: 1.78 ± 1.86	Frail: 50 Pre-frail: 32 Robust: 51 (133)	Community (USA)	Frailty phenotype (Fried et al., 2001)	Leng et al., 2011
		Plasma	mean ± SD [pg/mL]: 58.3 ± 10.2***	mean ± SD [pg/mL]: 43.4 ± 11.4	Frail: 12 Pre-frail: 31 Robust: 22 (65)	Community (USA)	Frailty phenotype (Fried et al., 2001)	Darvin et al., 2014
CRP	Increasing	Plasma	mean ± SD [mg/L]: 5.5 ± 9.8***	mean ± SD [mg/L]: 2.7 ± 4.0	Frail: 299 Pre-frail: 2,147 Robust: 2,289 (4,735)	Community (USA)	Frailty phenotype (Fried et al., 2001)	Walston et al., 2002 (Cardiovascular Health Study)
		Serum	mean ± SD [mg/L]: 4.2 ± 5.5***	mean ± SD [mg/L]: 3.0 ± 4.7	Frail: 234 Pre-frail: 1,854 Robust: 738 (2,826)	Community(USA)	Frailty phenotype (Fried et al., 2001)	Barzilay et al., 2007 (Cardiovascular Health Study)
		Serum	median (IQR) [mg/L]: 5.0 (3.0-13.0)*	median (IQR) [mg/L]: 3.0 (2.0-5.0)	Frail: 63 Pre-frail: 25 Robust: 22 (110)	Inpatients, outpatients and community (UK)	Frailty phenotype (Fried et al., 2001) + Frailty Index (Rockwood et al., 2005)†	Hubbard et al., 2009
TNF-α	Increasing	Plasma	mean ± SD [pg/mL]: 3.19 ± 2.68**	mean ± SD [pg/mL]: 1.50 ± 0.89	Frail: 63 Pre-frail: 25 Robust: 22 (110)	Inpatients, outpatients and community (UK)	Frailty phenotype (Fried et al., 2001) + Frailty Index (Rockwood et al., 2005)†	Hubbard et al., 2009
Factor VIII	Increasing	Plasma	mean ± SD [mg/dL]: 13,790 ± 4,480***	mean ± SD [mg/dL]: 11,860 ± 3,460	Frail: 299 Pre-frail: 2,147 Robust: 2,289 (4,735)	Community (USA)	Frailty phenotype (Fried et al., 2001)	Walston et al., 2002 (Cardiovascular Health Study)
Fibrinogen	Increasing	Plasma	mean ± SD [mg/dL]: 34,070 ± 7,860***	mean ± SD [mg/dL]: 31,330 ± 6,090	Frail: 299 Pre-frail: 2,147 Robust: 2,289 (4,735)	Community (USA)	Frailty phenotype (Fried et al., 2001)	Walston et al., 2002 (Cardiovascular Health Study)
		Plasma	mean ± SD [mg/dL]: 321.9 ± 61.4**	mean ± SD [mg/dL]: 309.4 ± 58.9	Frail: 234 Pre-frail: 1,854 Robust: 738 (2,826)	Community (USA)	Frailty phenotype (Fried et al., 2001)	Barzilay et al., 2007 (Cardiovascular Health Study)
		Plasma	mean ± SD [g/L]: 70.4 ± 17.5***	mean ± SD [g/L]: 40.6 ± 9.3	Frail: 12 Pre-frail: 31 Robust: 22 (65)	Community (USA)	Frailty phenotype (Fried et al., 2001)	Darvin et al., 2014
Transferrin	Increasing	Plasma	mean ± SD [ng/mL]: 58.3 ± 10.2***	mean ± SD [ng/mL]: 43.4 ± 11.4	Frail: 12 Pre-frail: 31 Robust: 22 (65)	Community (USA)	Frailty phenotype (Fried et al., 2001)	Darvin et al., 2014
CXCL10	Increasing	Total RNA from monocytes	mean ± SD (relative expression levels): 1.05 ± 0.88*	mean ± SD (relative expression levels): 0.53 ± 0.39	Frail: 16 Robust: 16 (32)	Community and outpatients (USA)	Frailty phenotype (Fried et al., 2001)	Qu et al., 2009
Neopterin	Increasing	Serum	mean ± SD [nM]: 10.53 ± 5.49***	mean ± SD [ng/mL]: 8.56 ± 1.80	Frail: 50 Pre-frail: 32 Robust: 51 (133)	Community (USA)	Frailty phenotype (Fried et al., 2001)	Leng et al., 2011

IL-6: interleukin 6; CRP: C-reactive protein; TNF-α: tumor necrosis factor alpha; CXCL10: C-X-C motif chemokine ligand 10; SD: standard deviation; IQR: interquartile range. Asterisks represent *p*-value (frail compared with robust individuals): *p<0.05; **p<0.01; ***p<0.001. Only statistically significant results were included. †Only values from patients categorized according to Fried´s frailty phenotype criteria were included for comparison purposes.

## Frailty in mice

The advances in the clinical characterization of frailty have been paralleled with preclinical studies in mice. These have facilitated the description of several scores to measure frailty in mice mostly based on Fried´s frailty phenotype or the FI, and also the generation of models to understand frailty and its underlying molecular basis, though this remains poorly understood [85].

### Scales to measure frailty domains in mice

The scores to measure frailty in mice are adapted from the scaling systems used in clinical assessments and include: 1) A method to quantify frailty based on deficit accumulation of 31 variables related to activity levels, hemodynamic status, body composition, and metabolism that exhibits key features of the FI in humans, and is subsequently called “Frailty index” [86, 87]. 2) A “clinically relevant frailty index for mice” including measurements of four out of five Fried´s criteria (weakness, low activity, poor endurance, and slowness, but not unintentional weight loss) by inverted-cling grip test, rotarod test, voluntary wheel running, and derived endurance scores from grip and rotarod tests [88]. Other measurement scales have been proposed following the five Fried´s criteria such as the “Valencia score”, which assesses several parameters (weight loss, running time and speed, grip strength, and motor coordination) that are equivalent to the key features described by Fried in humans [89]. In particular, body weight recording, treadmill test, grip strength test, and tightrope test were performed to evaluate the frailty status of mice.

Taking advantage of the mouse clinical frailty index, some biological biomarkers have been associated with frailty in mice such as lower levels of alanine aminotransferase (ALT), alkaline phosphatase (ALP), creatinine, and albumin. In addition to these serum biomarkers, reduced heart rate and heart rate variability were also correlated with frailty [90]. Notably, all of these measures had been previously related to frailty in humans.

Paralleling studies in humans, mice frailty seems to be reversible and different reports have shown that exercise intervention is able to delay the onset of frailty and the increase in the level of markers of oxidative stress [89, 91, 92]. These results support the idea that the mouse can be a good model to study the biology and mechanisms of frailty development, with as far as possible those related to physical frailty, and may also provide the opportunity to identify novel biomarkers that can be translated to humans.

Since there are some promising correlations between human and mouse frailty, it may be worthwhile to include tests to measure cognitive frailty in mice studies. In this regard, the “Valencia score” includes the tightrope test to resemble the low physical activity criterion [89], but this test also measures neuromuscular coordination activity [93], so it may be informative about aspects of the cognitive component of frailty. In agreement with this, other well-established cognitive tests such as the Elevated Plus Maze, the Open field, and the Morris Water Maze are starting to be used to measure cognitive frailty [90].

### Mouse models of frailty: relevance of oxidative stress and inflammation

Although still in early days, the study of aged mice, using the scales described above, and the generation of several mouse models, are beginning to unravel the underlying molecular mechanisms of frailty syndrome. In particular, oxidative stress and inflammation are mechanisms already associated with frailty in mice (Table 3).

**Table 3:** Main mouse models of frailty.

**ALTERED PATHWAY(S)**	**MOUSE MODEL**	**GENETIC MODIFICATION**	**FRAILTY PHENOTYPE**	**FRAILTY SCALE(S)**	**REFERENCE(S)**
OXIDATIVE STRESS	*G6PD-Tg*	Transgenic C57BL6/J-OlaHsd mice with overexpression of human G6PD	Increased median lifespan, improved neuromuscular fitness and glucose metabolism, higher NADPH levels, lower ROS-derived damage levels	Rotarod performance test	Nóbrega-Pereira et al., 2016
	Cu/Zn superoxide dismutase knockout (*Sod1KO*)	Deletion of *Sod1* gene	Decreased lifespan, weight loss, weakness, low physical activity and exhaustion, sarcopenia, higher oxidative damage, increased levels of NF-κB and pro-inflammatoy cytokines	Fried´s frailty phenotype	Deepa et al., 2017
INFLAMMATION	*IL-10^tm/tm^*	Deletion of *IL-10* gene	Decreased lifespan, reduced grip strength, increased IL-6 levels and other pro-inflammatory cytokines	Fried´s frailty phenotype	Walston et al., 2008 Ko et al., 2012
	*Nfκb1^−/−^*	Knockout of the *nfkb1* subunit of NF-κB	Reduced lifespan, body mass loss, impaired neuromuscular coordination, cachexia, increased IL-6 levels, cardiac hypertrophy, CD3^+^ infiltration	Tightrope test and aging-associated phenotypes	Jurk et al., 2014

G6PD: glucose-6-phosphate dehydrogenase; SOD1: superoxide dismutase 1; IL-10: interleukin 10; NF-κB: nuclear factor kappa B; ROS: reactive oxygen species; IL-6: interleukin 6; TNF-α: tumor necrosis factor alpha.

The *IL-10^tm/tm^* mouse model, which does not express the anti-inflammatory cytokine interleukin 10 (IL-10), was the first proposed mouse model of frailty [94] and it closely resembles human frail phenotypes, including chronic inflammation, with increased levels of cytokines such as IL-1β, IL-6, or TNF-α, muscle weakness, or impaired cardiac and vascular function [94–96]. Additional support for the role that inflammation has in frailty development comes from the *Nfκb1^−/−^* mice, in which the deficiency of nuclear factor kappa B (NF-κB) p105/p50 subunits results in chronic low grade inflammation and accelerated aging, and includes phenotypes closely related to frailty such as sarcopenia, body weight loss, and cardiac hypertrophy [97]. The elevated expression of some of the most relevant pro-inflammatory biomarkers of frailty in humans, such as IL-6 or TNF-α, in frail aged mice defined by the frailty scales cited before [92], further highlights the impact of inflammation as a biomarker and driver of frailty.

Studies in a transgenic mouse model with moderate overexpression of the antioxidant glucose-6-phosphate dehydrogenase (G6PD), the enzyme responsible for NADPH protection against oxidative damage, revealed that these mice exhibit increased resilience in response to age-associated decline of muscular and brain function [98]. Therefore, this suggests that a lower accumulation of oxidative damage delays the onset of physical and cognitive frailty in mice. Moreover, elevated oxidative stress has been associated with the premature incidence of frailty features as established in humans. Thus, the Cu/Zn superoxide dismutase knockout mouse (*Sod1KO*) exhibits four characteristics (weight loss, weakness, low physical activity, and exhaustion) that were originally used by Fried [99], underscoring the importance of oxidative stress as a mechanism of frailty development. In addition, these mice showed increased inflammation and sarcopenia, as well as alterations in pathways that have been linked to the etiology of frailty such as oxidative stress, mitochondrial dysfunction, and cell senescence, which have all been strongly related to human frailty [99]. Further evidence of the impact of oxidative stress in mice frailty comes from studies with the *IL-10^tm/tm^* mouse model, which presents elevated accumulation of oxidative damage in skeletal muscle [94, 100]. Together, these results highlight the value of these animal models in frailty research.

## CONCLUDING REMARKS

Many independent studies have revealed multiple putative biomarkers associated with frailty status at biological level. However, there are currently no accepted biomarkers that can be used as a reliable predictor of frailty across research and/or clinical fields. This is mainly attributable to limitations such as the heterogeneity of the tools, scales, and/or indices used to identify frail individuals, the limitations of some of the scales, the different age, sex, and characteristics across different populations, small sample sizes, limited longitudinal clinical studies, or the different techniques and cut-offs used for biomarker measurement.

Therefore, there is still a need for more robust biological biomarkers for accurate molecular identification of frail subjects. In this sense, it is now evident that efforts in the search of suitable frailty biomarkers should be directed to a panel of blood biomarkers rather than an assessment of individual molecules. This would contribute to better reflect the accumulation of damage and predict the risk of mortality in frail individuals, as recently highlighted [101]. Moreover, the majority of the proposed frailty biomarkers have been determined based on the frailty phenotype, which mostly measures reduced physical function. More studies should be conducted on the basis of deficit accumulation approach, also taking into account the cognitive domain of frailty as well as additional components.

Importantly, oxidative stress and inflammation are the most frequent pathways differentially expressed in frail compared to robust individuals [41, 72, 83]. Therefore, oxidative damage and inflammation-associated biomarkers exhibit the potential to become reliable frailty biomarkers; however, an exclusive biomarker of these processes that specifically identifies frailty in human samples has not yet been found, as they are also altered in aging and different age-associated diseases [52, 102]. Similarly, dysregulation of these processes is also common in frail mice. Moreover, the generation of mouse models such as *IL-10^tm/tm^*, *Sod1KO*, or transgenic mice for G6PD, which display multiple phenotypes of frailty, highlight the relevance of oxidative stress and inflammation in the pathophysiology of this syndrome. Indeed, results from clinical and preclinical studies have led to postulate the “free radical theory of frailty”, which proposes that oxidative damage is associated with frailty but not with chronological age itself [48]. Overall, the analysis in human studies and preclinical models is bringing to light the relevance of oxidative stress and inflammation as potential biomarkers and drivers of frailty.

## References

[1] McLaughlin SJ, Jette AM, Connell CM. An examination of healthy aging across a conceptual continuum: prevalence estimates, demographic patterns, and validity. J Gerontol A Biol Sci Med Sci. 2012; 67:783–89. 10.1093/gerona/glr234 22367432 PMC3536546

[2] Morley JE, Vellas B, van Kan GA, Anker SD, Bauer JM, Bernabei R, Cesari M, Chumlea WC, Doehner W, Evans J, Fried LP, Guralnik JM, Katz PR, et al. Frailty consensus: a call to action. J Am Med Dir Assoc. 2013; 14:392–97. 10.1016/j.jamda.2013.03.022 23764209 PMC4084863

[3] Collard RM, Boter H, Schoevers RA, Oude Voshaar RC. Prevalence of frailty in community-dwelling older persons: a systematic review. J Am Geriatr Soc. 2012; 60:1487–92. 10.1111/j.1532-5415.2012.04054.x 22881367

[4] Rodríguez-Mañas L, Féart C, Mann G, Viña J, Chatterji S, Chodzko-Zajko W, Gonzalez-Colaço Harmand M, Bergman H, Carcaillon L, Nicholson C, Scuteri A, Sinclair A, Pelaez M, et al, and FOD-CC group (Appendix 1). Searching for an operational definition of frailty: a delphi method based consensus statement: the frailty operative definition-consensus conference project. J Gerontol A Biol Sci Med Sci. 2013; 68:62–67. 10.1093/gerona/gls119 22511289 PMC3598366

[5] Xue QL. The frailty syndrome: definition and natural history. Clin Geriatr Med. 2011; 27:1–15. 10.1016/j.cger.2010.08.009 21093718 PMC3028599

[6] Rodríguez-Artalejo F, Rodríguez-Mañas L. The frailty syndrome in the public health agenda. J Epidemiol Community Health. 2014; 68:703–04. 10.1136/jech-2014-203863 24782415

[7] Cesari M, Prince M, Thiyagarajan JA, De Carvalho IA, Bernabei R, Chan P, Gutierrez-Robledo LM, Michel JP, Morley JE, Ong P, Rodriguez Manas L, Sinclair A, Won CW, et al. Frailty: an emerging public health priority. J Am Med Dir Assoc. 2016; 17:188–92. 10.1016/j.jamda.2015.12.016 26805753

[8] Theou O, Stathokostas L, Roland KP, Jakobi JM, Patterson C, Vandervoort AA, Jones GR. The effectiveness of exercise interventions for the management of frailty: a systematic review. J Aging Res. 2011; 2011:569194. 10.4061/2011/569194 21584244 PMC3092602

[9] Puts MT, Toubasi S, Andrew MK, Ashe MC, Ploeg J, Atkinson E, Ayala AP, Roy A, Rodríguez Monforte M, Bergman H, McGilton K. Interventions to prevent or reduce the level of frailty in community-dwelling older adults: a scoping review of the literature and international policies. Age Ageing. 2017; 46:383–92. 10.1093/ageing/afw247 28064173 PMC5405756

[10] Cadore EL, Rodríguez-Mañas L, Sinclair A, Izquierdo M. Effects of different exercise interventions on risk of falls, gait ability, and balance in physically frail older adults: a systematic review. Rejuvenation Res. 2013; 16:105–14. 10.1089/rej.2012.1397 23327448 PMC3634155

[11] Manal B, Suzana S, Singh DK. Nutrition and frailty: a review of clinical intervention studies. J Frailty Aging. 2015; 4:100–06. 10.14283/jfa.2015.49 27032052

[12] Tarazona-Santabalbina FJ, Gómez-Cabrera MC, Pérez-Ros P, Martínez-Arnau FM, Cabo H, Tsaparas K, Salvador-Pascual A, Rodriguez-Mañas L, Viña J. A multicomponent exercise intervention that reverses frailty and improves cognition, emotion, and social networking in the community-dwelling frail elderly: a randomized clinical trial. J Am Med Dir Assoc. 2016; 17:426–33. 10.1016/j.jamda.2016.01.019 26947059

[13] Cameron ID, Fairhall N, Langron C, Lockwood K, Monaghan N, Aggar C, Sherrington C, Lord SR, Kurrle SE. A multifactorial interdisciplinary intervention reduces frailty in older people: randomized trial. BMC Med. 2013; 11:65. 10.1186/1741-7015-11-65 23497404 PMC3751685

[14] Walston J, Buta B, Xue QL. Frailty screening and interventions: considerations for clinical practice. Clin Geriatr Med. 2018; 34:25–38. 10.1016/j.cger.2017.09.004 29129215 PMC5726589

[15] Fried LP, Tangen CM, Walston J, Newman AB, Hirsch C, Gottdiener J, Seeman T, Tracy R, Kop WJ, Burke G, McBurnie MA, and Cardiovascular Health Study Collaborative Research Group. Frailty in older adults: evidence for a phenotype. J Gerontol A Biol Sci Med Sci. 2001; 56:M146–56. 10.1093/gerona/56.3.m146 11253156

[16] Mitnitski AB, Mogilner AJ, Rockwood K. Accumulation of deficits as a proxy measure of aging. ScientificWorldJournal. 2001; 1:323–36. 10.1100/tsw.2001.58 12806071 PMC6084020

[17] Abellan van Kan G, Rolland YM, Morley JE, Vellas B. Frailty: toward a clinical definition. J Am Med Dir Assoc. 2008; 9:71–72. 10.1016/j.jamda.2007.11.005 18261696

[18] Gobbens RJ, van Assen MA, Luijkx KG, Wijnen-Sponselee MT, Schols JM. The tilburg frailty indicator: psychometric properties. J Am Med Dir Assoc. 2010; 11:344–55. 10.1016/j.jamda.2009.11.003 20511102

[19] Studenski S, Hayes RP, Leibowitz RQ, Bode R, Lavery L, Walston J, Duncan P, Perera S. Clinical global impression of change in physical frailty: development of a measure based on clinical judgment. J Am Geriatr Soc. 2004; 52:1560–66. 10.1111/j.1532-5415.2004.52423.x 15341562

[20] Rockwood K, Song X, MacKnight C, Bergman H, Hogan DB, McDowell I, Mitnitski A. A global clinical measure of fitness and frailty in elderly people. CMAJ. 2005; 173:489–95. 10.1503/cmaj.050051 16129869 PMC1188185

[21] Vellas B, Balardy L, Gillette-Guyonnet S, Abellan Van Kan G, Ghisolfi-Marque A, Subra J, Bismuth S, Oustric S, Cesari M. Looking for frailty in community-dwelling older persons: the gérontopôle frailty screening tool (GFST). J Nutr Health Aging. 2013; 17:629–31. 10.1007/s12603-013-0363-623933875

[22] Rodríguez-Mañas L. Use of biomarkers. J Frailty Aging. 2015; 4:125–28. 10.14283/jfa.2015.46 27030939

[23] Erusalimsky JD, Grillari J, Grune T, Jansen-Duerr P, Lippi G, Sinclair AJ, Tegnér J, Viña J, Durrance-Bagale A, Miñambres R, Viegas M, Rodríguez-Mañas L, and FRAILOMIC Consortium. In search of ‘Omics’-based biomarkers to predict risk of frailty and its consequences in older individuals: the FRAILOMIC initiative. Gerontology. 2016; 62:182–90. 10.1159/000435853 26227153

[24] Cardoso AL, Fernandes A, Aguilar-Pimentel JA, de Angelis MH, Guedes JR, Brito MA, Ortolano S, Pani G, Athanasopoulou S, Gonos ES, Schosserer M, Grillari J, Peterson P, et al. Towards frailty biomarkers: candidates from genes and pathways regulated in aging and age-related diseases. Ageing Res Rev. 2018; 47:214–77. 10.1016/j.arr.2018.07.004 30071357

[25] Kelaiditi E, Cesari M, Canevelli M, van Kan GA, Ousset PJ, Gillette-Guyonnet S, Ritz P, Duveau F, Soto ME, Provencher V, Nourhashemi F, Salvà A, Robert P, et al, and IANA/IAGG. Cognitive frailty: rational and definition from an (I.A.N.A./I.A.G.G.) international consensus group. J Nutr Health Aging. 2013; 17:726–34. 10.1007/s12603-013-0367-224154642

[26] Panza F, Solfrizzi V, Barulli MR, Santamato A, Seripa D, Pilotto A, Logroscino G. Cognitive frailty: a systematic review of epidemiological and neurobiological evidence of an age-related clinical condition. Rejuvenation Res. 2015; 18:389–412. 10.1089/rej.2014.1637 25808052

[27] Fitten LJ. Psychological frailty in the aging patient. Nestle Nutr Inst Workshop Ser. 2015; 83:45–53. 10.1159/000382060 26484526

[28] Hoogendijk EO, Afilalo J, Ensrud KE, Kowal P, Onder G, Fried LP. Frailty: implications for clinical practice and public health. Lancet. 2019; 394:1365–75. 10.1016/S0140-6736(19)31786-6 31609228

[29] Lorenzo-López L, Maseda A, de Labra C, Regueiro-Folgueira L, Rodríguez-Villamil JL, Millán-Calenti JC. Nutritional determinants of frailty in older adults: a systematic review. BMC Geriatr. 2017; 17:108. 10.1186/s12877-017-0496-2 28506216 PMC5433026

[30] Morley JE. The new geriatric giants. Clin Geriatr Med. 2017; 33. 10.1016/j.cger.2017.05.001 28689574

[31] Stenholm S, Ferrucci L, Vahtera J, Hoogendijk EO, Huisman M, Pentti J, Lindbohm JV, Bandinelli S, Guralnik JM, Kivimäki M. Natural course of frailty components in people who develop frailty syndrome: evidence from two cohort studies. J Gerontol A Biol Sci Med Sci. 2019; 74:667–74. 10.1093/gerona/gly132 30084927 PMC6477647

[32] Dent E, Morley JE, Cruz-Jentoft AJ, Arai H, Kritchevsky SB, Guralnik J, Bauer JM, Pahor M, Clark BC, Cesari M, Ruiz J, Sieber CC, Aubertin-Leheudre M, et al. International Clinical Practice Guidelines for Sarcopenia (ICFSR): Screening, Diagnosis and Management. J Nutr Health Aging. 2018; 22:1148–1161. 10.1007/s12603-018-1139-930498820

[33] Dent E, Morley JE, Cruz-Jentoft AJ, Woodhouse L, Rodríguez-Mañas L, Fried LP, Woo J, Aprahamian I, Sanford A, Lundy J, Landi F, Beilby J, Martin FC, et al. Physical frailty: ICFSR international clinical practice guidelines for identification and management. J Nutr Health Aging. 2019; 23:771–87. 10.1007/s12603-019-1273-z 31641726 PMC6800406

[34] Fried LP, Ferrucci L, Darer J, Williamson JD, Anderson G. Untangling the concepts of disability, frailty, and comorbidity: implications for improved targeting and care. J Gerontol A Biol Sci Med Sci. 2004; 59:255–63. 10.1093/gerona/59.3.m255 15031310

[35] Cesari M, Landi F, Vellas B, Bernabei R, Marzetti E. Sarcopenia and physical frailty: two sides of the same coin. Front Aging Neurosci. 2014; 6:192. 10.3389/fnagi.2014.00192 25120482 PMC4112807

[36] Davies B, García F, Ara I, Artalejo FR, Rodriguez-Mañas L, Walter S. Relationship between sarcopenia and frailty in the toledo study of healthy aging: a population based cross-sectional study. J Am Med Dir Assoc. 2018; 19:282–86. 10.1016/j.jamda.2017.09.014 29079029

[37] Robertson DA, Savva GM, Kenny RA. Frailty and cognitive impairment—a review of the evidence and causal mechanisms. Ageing Res Rev. 2013; 12:840–51. 10.1016/j.arr.2013.06.004 23831959

[38] Malmstrom TK, Morley JE. Frailty and cognition: linking two common syndromes in older persons. J Nutr Health Aging. 2013; 17:723–25. 10.1007/s12603-013-0395-y24154641

[39] Sugimoto T, Sakurai T, Ono R, Kimura A, Saji N, Niida S, Toba K, Chen LK, Arai H. Epidemiological and clinical significance of cognitive frailty: a mini review. Ageing Res Rev. 2018; 44:1–7. 10.1016/j.arr.2018.03.002 29544875

[40] Facal D, Maseda A, Pereiro AX, Gandoy-Crego M, Lorenzo-López L, Yanguas J, Millán-Calenti JC. Cognitive frailty: a conceptual systematic review and an operational proposal for future research. Maturitas. 2019; 121:48–56. 10.1016/j.maturitas.2018.12.006 30704565

[41] Sargent L, Nalls M, Starkweather A, Hobgood S, Thompson H, Amella EJ, Singleton A. Shared biological pathways for frailty and cognitive impairment: a systematic review. Ageing Res Rev. 2018; 47:149–58. 10.1016/j.arr.2018.08.001 30102995 PMC6376483

[42] Woods AJ, Cohen RA, Pahor M. Cognitive frailty: frontiers and challenges. J Nutr Health Aging. 2013; 17:741–43. 10.1007/s12603-013-0398-8 24154645 PMC4471842

[43] Brinkley TE, Leng X, Miller ME, Kitzman DW, Pahor M, Berry MJ, Marsh AP, Kritchevsky SB, Nicklas BJ. Chronic inflammation is associated with low physical function in older adults across multiple comorbidities. J Gerontol A Biol Sci Med Sci. 2009; 64:455–61. 10.1093/gerona/gln038 19196644 PMC2657165

[44] Panza F, Lozupone M, Solfrizzi V, Stallone R, Bellomo A, Greco A, Daniele A, Seripa D, Logroscino G. Cognitive frailty: a potential target for secondary prevention of dementia. Expert Opin Drug Metab Toxicol. 2017; 13:1023–27. 10.1080/17425255.2017.1372424 28849681

[45] Rodríguez Mañas L. Determinants of frailty and longevity: are they the same ones? Nestle Nutr Inst Workshop Ser. 2015; 83:29–39. 10.1159/000382057 26485702

[46] Kane AE, Sinclair DA. Frailty biomarkers in humans and rodents: current approaches and future advances. Mech Ageing Dev. 2019; 180:117–28. 10.1016/j.mad.2019.03.007 31002925 PMC6581034

[47] Soysal P, Isik AT, Carvalho AF, Fernandes BS, Solmi M, Schofield P, Veronese N, Stubbs B. Oxidative stress and frailty: a systematic review and synthesis of the best evidence. Maturitas. 2017; 99:66–72. 10.1016/j.maturitas.2017.01.006 28364871

[48] Viña J, Borras C, Gomez-Cabrera MC. A free radical theory of frailty. Free Radic Biol Med. 2018; 124:358–63. 10.1016/j.freeradbiomed.2018.06.028 29958933

[49] Liu CK, Lyass A, Larson MG, Massaro JM, Wang N, D’Agostino RB Sr, Benjamin EJ, Murabito JM. Biomarkers of oxidative stress are associated with frailty: the framingham offspring study. Age (Dordr). 2016; 38:1. 10.1007/s11357-015-9864-z 26695510 PMC5005887

[50] Packard CJ, O'Reilly DS, Caslake MJ, McMahon AD, Ford I, Cooney J, Macphee CH, Suckling KE, Krishna M, Wilkinson FE, Rumley A, Lowe GD. Lipoprotein-associated phospholipase A2 as an independent predictor of coronary heart disease. West of Scotland Coronary Prevention Study Group. N Engl J Med. 2000; 343:1148–55. 11036120 10.1056/NEJM200010193431603

[51] Syslová K, Böhmová A, Mikoška M, Kuzma M, Pelclová D, Kačer P. Multimarker screening of oxidative stress in aging. Oxid Med Cell Longev. 2014; 2014:562860. 10.1155/2014/562860 25147595 PMC4124763

[52] Namioka N, Hanyu H, Hirose D, Hatanaka H, Sato T, Shimizu S. Oxidative stress and inflammation are associated with physical frailty in patients with alzheimer’s disease. Geriatr Gerontol Int. 2017; 17:913–18. 10.1111/ggi.12804 27296166

[53] Czerska M, Zieliński M, Gromadzińska J. Isoprostanes - a novel major group of oxidative stress markers. Int J Occup Med Environ Health. 2016; 29:179–90. 10.13075/ijomeh.1896.00596 26670350

[54] Wu IC, Shiesh SC, Kuo PH, Lin XZ. High oxidative stress is correlated with frailty in elderly chinese. J Am Geriatr Soc. 2009; 57:1666–71. 10.1111/j.1532-5415.2009.02392.x 19682113

[55] Cesarone MR, Belcaro G, Carratelli M, Cornelli U, De Sanctis MT, Incandela L, Barsotti A, Terranova R, Nicolaides A. A simple test to monitor oxidative stress. Int Angiol. 1999; 18:127–30. 10424368

[56] Saum KU, Dieffenbach AK, Jansen EH, Schöttker B, Holleczek B, Hauer K, Brenner H. Association between oxidative stress and frailty in an elderly german population: results from the ESTHER cohort study. Gerontology. 2015; 61:407–15. 10.1159/000380881 25924722

[57] Hirata Y, Yamamoto E, Tokitsu T, Kusaka H, Fujisue K, Kurokawa H, Sugamura K, Maeda H, Tsujita K, Kaikita K, Hokimoto S, Sugiyama S, Ogawa H. Reactive oxygen metabolites are closely associated with the diagnosis and prognosis of coronary artery disease. J Am Heart Assoc. 2015; 4:e001451. 10.1161/JAHA.114.001451 25630910 PMC4345871

[58] Inglés M, Gambini J, Carnicero JA, García-García FJ, Rodríguez-Mañas L, Olaso-González G, Dromant M, Borrás C, Viña J. Oxidative stress is related to frailty, not to age or sex, in a geriatric population: lipid and protein oxidation as biomarkers of frailty. J Am Geriatr Soc. 2014; 62:1324–28. 10.1111/jgs.12876 24962132

[59] Howard C, Ferrucci L, Sun K, Fried LP, Walston J, Varadhan R, Guralnik JM, Semba RD. Oxidative protein damage is associated with poor grip strength among older women living in the community. J Appl Physiol (1985). 2007; 103:17–20. 10.1152/japplphysiol.00133.2007 17379753 PMC2646087

[60] Semba RD, Ferrucci L, Sun K, Walston J, Varadhan R, Guralnik JM, Fried LP. Oxidative stress and severe walking disability among older women. Am J Med. 2007; 120:1084–89. 10.1016/j.amjmed.2007.07.028 18060930 PMC2423489

[61] de Gonzalo-Calvo D, de Luxán-Delgado B, Rodríguez-González S, García-Macia M, Suárez FM, Solano JJ, Rodríguez-Colunga MJ, Coto-Montes A. Oxidative protein damage is associated with severe functional dependence among the elderly population: a principal component analysis approach. J Gerontol A Biol Sci Med Sci. 2012; 67:663–70. 10.1093/gerona/glr215 22179933

[62] Nielsen F, Mikkelsen BB, Nielsen JB, Andersen HR, Grandjean P. Plasma malondialdehyde as biomarker for oxidative stress: reference interval and effects of life-style factors. Clin Chem. 1997; 43:1209–14. 9216458

[63] Serviddio G, Romano AD, Greco A, Rollo T, Bellanti F, Altomare E, Vendemiale G. Frailty syndrome is associated with altered circulating redox balance and increased markers of oxidative stress. Int J Immunopathol Pharmacol. 2009; 22:819–27. 10.1177/039463200902200328 19822098

[64] Goulet ED, Hassaine A, Dionne IJ, Gaudreau P, Khalil A, Fulop T, Shatenstein B, Tessier D, Morais JA. Frailty in the elderly is associated with insulin resistance of glucose metabolism in the postabsorptive state only in the presence of increased abdominal fat. Exp Gerontol. 2009; 44:740–44. 10.1016/j.exger.2009.08.008 19723576

[65] Gil P, Fariñas F, Casado A, López-Fernández E. Malondialdehyde: a possible marker of ageing. Gerontology. 2002; 48:209–14. 10.1159/000058352 12053109

[66] Hu ML. Measurement of protein thiol groups and glutathione in plasma. Methods Enzymol. 1994; 233:380–85. 10.1016/s0076-6879(94)33044-1 8015473

[67] El Assar M, Angulo J, Carnicero JA, Walter S, García-García FJ, López-Hernández E, Sánchez-Puelles JM, Rodríguez-Mañas L. Frailty is associated with lower expression of genes involved in cellular response to stress: results from the toledo study for healthy aging. J Am Med Dir Assoc. 2017; 18:734.e1–734.e7. 10.1016/j.jamda.2017.04.019 28647579

[68] Ble A, Cherubini A, Volpato S, Bartali B, Walston JD, Windham BG, Bandinelli S, Lauretani F, Guralnik JM, Ferrucci L. Lower plasma vitamin E levels are associated with the frailty syndrome: the InCHIANTI study. J Gerontol A Biol Sci Med Sci. 2006; 61:278–83. 10.1093/gerona/61.3.278 16567378

[69] Pilleron S, Rajaobelina K, Tabue Teguo M, Dartigues JF, Helmer C, Delcourt C, Rigalleau V, Féart C. Accumulation of advanced glycation end products evaluated by skin autofluorescence and incident frailty in older adults from the bordeaux three-city cohort. PLoS One. 2017; 12:e0186087. 10.1371/journal.pone.0186087 29040310 PMC5645102

[70] Semba RD, Bartali B, Zhou J, Blaum C, Ko CW, Fried LP. Low serum micronutrient concentrations predict frailty among older women living in the community. J Gerontol A Biol Sci Med Sci. 2006; 61:594–99. 10.1093/gerona/61.6.594 16799142

[71] Michelon E, Blaum C, Semba RD, Xue QL, Ricks MO, Fried LP. Vitamin and carotenoid status in older women: associations with the frailty syndrome. J Gerontol A Biol Sci Med Sci. 2006; 61:600–07. 10.1093/gerona/61.6.600 16799143

[72] Rietman ML, Spijkerman AM, Wong A, van Steeg H, Bürkle A, Moreno-Villanueva M, Sindlinger T, Franceschi C, Grubeck-Loebenstein B, Bernhardt J, Slagboom PE, Toussaint O, Debacq-Chainiaux F, et al. Antioxidants linked with physical, cognitive and psychological frailty: analysis of candidate biomarkers and markers derived from the MARK-AGE study. Mech Ageing Dev. 2019; 177:135–43. 10.1016/j.mad.2018.04.007 29719199

[73] Franceschi C, Bonafè M, Valensin S, Olivieri F, De Luca M, Ottaviani E, De Benedictis G. Inflamm-aging. An evolutionary perspective on immunosenescence. Ann N Y Acad Sci. 2000; 908:244–54. 10.1111/j.1749-6632.2000.tb06651.x 10911963

[74] Ferrucci L, Fabbri E. Inflammageing: chronic inflammation in ageing, cardiovascular disease, and frailty. Nat Rev Cardiol. 2018; 15:505–22. 10.1038/s41569-018-0064-2 30065258 PMC6146930

[75] Leng S, Chaves P, Koenig K, Walston J. Serum interleukin-6 and hemoglobin as physiological correlates in the geriatric syndrome of frailty: a pilot study. J Am Geriatr Soc. 2002; 50:1268–71. 10.1046/j.1532-5415.2002.50315.x 12133023

[76] Walston J, McBurnie MA, Newman A, Tracy RP, Kop WJ, Hirsch CH, Gottdiener J, Fried LP, and Cardiovascular Health Study. Frailty and activation of the inflammation and coagulation systems with and without clinical comorbidities: results from the cardiovascular health study. Arch Intern Med. 2002; 162:2333–41. 10.1001/archinte.162.20.2333 12418947

[77] Barzilay JI, Blaum C, Moore T, Xue QL, Hirsch CH, Walston JD, Fried LP. Insulin resistance and inflammation as precursors of frailty: the cardiovascular health study. Arch Intern Med. 2007; 167:635–41. 10.1001/archinte.167.7.635 17420420

[78] Darvin K, Randolph A, Ovalles S, Halade D, Breeding L, Richardson A, Espinoza SE. Plasma protein biomarkers of the geriatric syndrome of frailty. J Gerontol A Biol Sci Med Sci. 2014; 69:182–86. 10.1093/gerona/glt183 24285743 PMC4038243

[79] Soysal P, Stubbs B, Lucato P, Luchini C, Solmi M, Peluso R, Sergi G, Isik AT, Manzato E, Maggi S, Maggio M, Prina AM, Cosco TD, et al. Inflammation and frailty in the elderly: A systematic review and meta-analysis. Ageing Res Rev. 2016; 31:1–8. 10.1016/j.arr.2016.08.006 27592340

[80] Collerton J, Martin-Ruiz C, Davies K, Hilkens CM, Isaacs J, Kolenda C, Parker C, Dunn M, Catt M, Jagger C, von Zglinicki T, Kirkwood TB. Frailty and the role of inflammation, immunosenescence and cellular ageing in the very old: cross-sectional findings from the newcastle 85+ study. Mech Ageing Dev. 2012; 133:456–66. 10.1016/j.mad.2012.05.005 22663935

[81] Qu T, Yang H, Walston JD, Fedarko NS, Leng SX. Upregulated monocytic expression of CXC chemokine ligand 10 (CXCL-10) and its relationship with serum interleukin-6 levels in the syndrome of frailty. Cytokine. 2009; 46:319–24. 10.1016/j.cyto.2009.02.015 19342252 PMC3159181

[82] Leng SX, Tian X, Matteini A, Li H, Hughes J, Jain A, Walston JD, Fedarko NS. IL-6-independent association of elevated serum neopterin levels with prevalent frailty in community-dwelling older adults. Age Ageing. 2011; 40:475–81. 10.1093/ageing/afr047 21586579 PMC3114624

[83] Ruan Q, D'Onofrio G, Sancarlo D, Greco A, Lozupone M, Seripa D, Panza F, Yu Z. Emerging biomarkers and screening for cognitive frailty. Aging Clin Exp Res. 2017; 29:1075–86. 10.1007/s40520-017-0741-8 28260159

[84] Solfrizzi V, Scafato E, Lozupone M, Seripa D, Giannini M, Sardone R, Bonfiglio C, Abbrescia DI, Galluzzo L, Gandin C, Baldereschi M, Di Carlo A, Inzitari D, et al, and Italian Longitudinal Study on Aging Working Group. Additive role of a potentially reversible cognitive frailty model and inflammatory state on the risk of disability: the italian longitudinal study on aging. Am J Geriatr Psychiatry. 2017; 25:1236–48. 10.1016/j.jagp.2017.05.018 28689645

[85] von Zglinicki T, Varela Nieto I, Brites D, Karagianni N, Ortolano S, Georgopoulos S, Cardoso AL, Novella S, Lepperdinger G, Trendelenburg AU, van Os R. Frailty in mouse ageing: a conceptual approach. Mech Ageing Dev. 2016; 160:34–40. 10.1016/j.mad.2016.07.004 27443148

[86] Parks RJ, Fares E, Macdonald JK, Ernst MC, Sinal CJ, Rockwood K, Howlett SE. A procedure for creating a frailty index based on deficit accumulation in aging mice. J Gerontol A Biol Sci Med Sci. 2012; 67:217–27. 10.1093/gerona/glr193 22021390

[87] Whitehead JC, Hildebrand BA, Sun M, Rockwood MR, Rose RA, Rockwood K, Howlett SE. A clinical frailty index in aging mice: comparisons with frailty index data in humans. J Gerontol A Biol Sci Med Sci. 2014; 69:621–32. 10.1093/gerona/glt136 24051346 PMC4022099

[88] Liu H, Graber TG, Ferguson-Stegall L, Thompson LV. Clinically relevant frailty index for mice. J Gerontol A Biol Sci Med Sci. 2014; 69:1485–91. 10.1093/gerona/glt188 24336799 PMC4271019

[89] Gomez-Cabrera MC, Garcia-Valles R, Rodriguez-Mañas L, Garcia-Garcia FJ, Olaso-Gonzalez G, Salvador-Pascual A, Tarazona-Santabalbina FJ, Viña J. A new frailty score for experimental animals based on the clinical phenotype: inactivity as a model of frailty. J Gerontol A Biol Sci Med Sci. 2017; 72:885–91. 10.1093/gerona/glw337 28329258

[90] Kane AE, Huizer-Pajkos A, Mach J, Mitchell SJ, de Cabo R, Le Couteur DG, Howlett SE, Hilmer SN. A comparison of two mouse frailty assessment tools. J Gerontol A Biol Sci Med Sci. 2017; 72:904–09. 10.1093/gerona/glx009 28549083 PMC5861875

[91] Garcia-Valles R, Gomez-Cabrera MC, Rodriguez-Mañas L, Garcia-Garcia FJ, Diaz A, Noguera I, Olaso-Gonzalez G, Viña J. Life-long spontaneous exercise does not prolong lifespan but improves health span in mice. Longev Healthspan. 2013; 2:14. 10.1186/2046-2395-2-14 24472376 PMC3922914

[92] Banga S, Heinze-Milne SD, Howlett SE. Rodent models of frailty and their application in preclinical research. Mech Ageing Dev. 2019; 179:1–10. 10.1016/j.mad.2019.01.008 30703384

[93] Miquel J, Blasco M. A simple technique for evaluation of vitality loss in aging mice, by testing their muscular coordination and vigor. Exp Gerontol. 1978; 13:389–96. 10.1016/0531-5565(78)90049-9 738379

[94] Walston J, Fedarko N, Yang H, Leng S, Beamer B, Espinoza S, Lipton A, Zheng H, Becker K. The physical and biological characterization of a frail mouse model. J Gerontol A Biol Sci Med Sci. 2008; 63:391–98. 10.1093/gerona/63.4.391 18426963 PMC5903428

[95] Ko F, Yu Q, Xue QL, Yao W, Brayton C, Yang H, Fedarko N, Walston J. Inflammation and mortality in a frail mouse model. Age (Dordr). 2012; 34:705–15. 10.1007/s11357-011-9269-6 21633802 PMC3337927

[96] Sikka G, Miller KL, Steppan J, Pandey D, Jung SM, Fraser CD 3rd, Ellis C, Ross D, Vandegaer K, Bedja D, Gabrielson K, Walston JD, Berkowitz DE, Barouch LA. Interleukin 10 knockout frail mice develop cardiac and vascular dysfunction with increased age. Exp Gerontol. 2013; 48:128–35. 10.1016/j.exger.2012.11.001 23159957 PMC3744178

[97] Jurk D, Wilson C, Passos JF, Oakley F, Correia-Melo C, Greaves L, Saretzki G, Fox C, Lawless C, Anderson R, Hewitt G, Pender SL, Fullard N, et al. Chronic inflammation induces telomere dysfunction and accelerates ageing in mice. Nat Commun. 2014; 2:4172. 10.1038/ncomms5172 24960204 PMC4090717

[98] Nóbrega-Pereira S, Fernandez-Marcos PJ, Brioche T, Gomez-Cabrera MC, Salvador-Pascual A, Flores JM, Viña J, Serrano M. G6PD protects from oxidative damage and improves healthspan in mice. Nat Commun. 2016; 7:10894. 10.1038/ncomms10894 26976705 PMC4796314

[99] Deepa SS, Bhaskaran S, Espinoza S, Brooks SV, McArdle A, Jackson MJ, Van Remmen H, Richardson A. A new mouse model of frailty: the cu/zn superoxide dismutase knockout mouse. Geroscience. 2017; 39:187–98. 10.1007/s11357-017-9975-9 28409332 PMC5411367

[100] Abadir P, Ko F, Marx R, Powell L, Kieserman E, Yang H, Walston J. Co-localization of macrophage inhibitory factor and nix in skeletal muscle of the aged male interleukin 10 null mouse. J Frailty Aging. 2017; 6:118–21. 10.14283/jfa.2017.18 28721426 PMC5664939

[101] Mitnitski A, Collerton J, Martin-Ruiz C, Jagger C, von Zglinicki T, Rockwood K, Kirkwood TB. Age-related frailty and its association with biological markers of ageing. BMC Med. 2015; 13:161. 10.1186/s12916-015-0400-x 26166298 PMC4499935

[102] Weber D, Stuetz W, Toussaint O, Debacq-Chainiaux F, Dollé ME, Jansen E, Gonos ES, Franceschi C, Sikora E, Hervonen A, Breusing N, Sindlinger T, Moreno-Villanueva M, et al. Associations between specific redox biomarkers and age in a large european cohort: the MARK-AGE project. Oxid Med Cell Longev. 2017; 2017:1401452. 10.1155/2017/1401452 28804532 PMC5539926

